# Responding to symptoms suggestive of lung cancer: a qualitative interview study

**DOI:** 10.1136/bmjresp-2014-000067

**Published:** 2014-12-11

**Authors:** Linda Birt, Nicky Hall, Jon Emery, Jon Banks, Katie Mills, Margaret Johnson, Willie Hamilton, Fiona M Walter

**Affiliations:** 1 Department of Public Health & Primary Care, University of Cambridge, Cambridge, UK; 2 School of Medicine, Pharmacy & Health, Durham University, Durham, UK; 3 General Practice & Primary Care Academic Centre, University of Melbourne, Melbourne, Australia; 4 School of Social & Community Medicine, University of Bristol, Bristol, UK; 5 Lay Member of Study Steering Committee; 6 University of Exeter, Exeter, UK

**Keywords:** Lung Cancer, Psychology

## Abstract

**Background:**

Late diagnosis of lung cancer can impact on survival rates. Patients delay seeking help for a number of reasons. This study explored symptom appraisal and help-seeking decisions among patients referred to specialist respiratory services with symptoms suggestive of lung cancer.

**Methods:**

In-depth qualitative interviews with patients as soon as possible after referral, ideally before diagnosis and mainly within 10 weeks, explored factors impacting on their pathways prior to referral. Framework analysis, underpinned by the Model of Pathways to Treatment, was used to explore the data with particular focus on patients’ beliefs and experiences, disease factors and healthcare professional influences.

**Results:**

35 patients were interviewed (aged 41–88 years, 15 women, 17 with lung cancer). All described similar presenting symptoms and triggers to seek help. Appraisal of symptoms was influenced by whether they had a lung comorbidity; seriousness of symptoms was interpreted within the context of previous illness experiences. Help-seeking was triggered when: symptoms failed to respond as expected; there was an increased awareness of symptoms of lung cancer; the public nature of a cough meant others were able to endorse help-seeking. Almost half visited the general practitioner (GP) two or more times before referral; during this period they reinterpreted initial symptoms and appraised new symptoms. The meaning given to symptoms changed over time and many became increasingly concerned they may have lung cancer. The GP played a role in ensuring timely further help-seeking but often there was little guidance on how to monitor symptoms or when to reconsult.

**Conclusions:**

Patients diagnosed with and without lung cancer had similar symptom pathways. Findings provide guidance for lung cancer awareness campaigns on the importance of social networks in endorsing patient help-seeking. The importance of appropriate advice, monitoring and safety-netting procedures by GPs for people presenting with symptoms suggestive of lung cancer is also highlighted.

**Key messages**

- We believe this study is the first to compare the appraisal and help-seeking experiences of patients with symptoms indicative of lung cancer (such as cough, dyspnoea, haemoptysis, chest and back pain) between people subsequently diagnosed with lung cancer and people diagnosed with other non-cancer conditions.
- The study was guided by the Aarhus statement recommendations on improving design and reporting of studies on early cancer diagnosis.
- Recruiting at the time of referral to specialist respiratory services and interviewing patients before or close to diagnosis reduced risk of post hoc rationalisation and recall bias.
- Almost half of the study group visited the general practitioner (GP) two or more times before an appropriate investigation or referral was made, enabling us to report on the patient perception of GP advice on symptom monitoring and on when to reconsult.

## Introduction

Lung cancer is the most common cause of cancer death in the UK. While there has been a fall in incidence in men, there has been a slow, steady increase of cases in women. It continues to have one of the poorest 5-year relative survival rates of all cancers,^1^ only 7.8% for men and 9.3% for women.^2^ Most cases of lung cancer present symptomatically, and poor survival rates are primarily due to later stage disease, the biology of the disease, lack of screening and fewer treatment options.^3^ ^4^ Diagnosis in primary care is challenging as the majority of patients who present to their general practitioner (GP) with respiratory symptoms will not have lung cancer.^5^ Survival rates in the UK from lung cancer are poorer than in other European countries^6^ and it may be that negative beliefs about barriers to symptomatic presentation contribute to this.^7^ Understanding how patients recognise possible signs of lung cancer and the decisions they make about seeking help for their symptoms can inform the development of interventions to reduce the time to diagnose lung cancer and potentially improve survival.

The timeliness of patient help-seeking for potential cancer symptoms is influenced by a number of factors. A UK population survey, using the validated Cancer Awareness Measure, identified four key barriers to timely help-seeking: *perceived service barriers*, that is, difficulty getting an appointment; *practical barriers*, that is, lacking the time or transport to attend the consultation; *emotional barriers* that is, fear of receiving bad news, and *failing to interpret the symptom/s as requiring medical attention.*^8^ Studies explicitly investigating the help-seeking experiences of people diagnosed with lung cancer have reported similar barriers.^9–15^ Furthermore, the recognition of new respiratory symptoms is particularly difficult for patients who have a lung comorbidity such as chronic obstructive pulmonary disease (COPD).^5^ Many patients at risk of lung cancer have a history of smoking, which can further prolong help-seeking owing to perceived risks of being stigmatised and of not being worthy of medical help.^10^

Raising awareness of cancer symptoms may help to promote timely help-seeking. Public health campaigns such as the ‘Be Clear on Cancer’ lung cancer campaign^16^ have been used to raise awareness of the signs of lung cancer. At a community level, there is evidence of increased awareness of lung cancer symptoms and increase in referrals to specialist respiratory services during similar campaigns.^17^ ^18^ However, understanding the possible cause of a symptom does not always directly translate into seeking a consultation to discuss such symptoms.^19^ People have to make complex decisions about when it is appropriate to seek help, and in part these decisions are influenced by perception of personal risk.^20^ Decisions to seek help are also shaped by public and professional perception of what is a reasonable time to wait for symptoms to resolve spontaneously. While there is general consensus that ‘red flag symptoms’ such as haemoptysis^21^ should be presented and referred urgently, National Institute for Clinical Evidence (NICE) recommends that other respiratory symptoms such as cough and dyspnoea should have been present for at least 3 weeks before investigation by chest X-ray.^22^ Therefore, it is important that we more fully understand the reasoning behind patients’ help-seeking decisions for a range of respiratory symptoms in order that interventions, particularly those aimed at promoting presentation of symptoms, can be developed to improve timeliness of help-seeking. To date, studies exploring patient appraisal and help-seeking for symptoms suggestive of lung cancer have only reported the experiences of those diagnosed with lung cancer, and have interviewed or surveyed patients often several months or years after diagnosis. These studies may be biased by post hoc rationalisation and recall bias. Patients are more likely to recall their appraisal and help-seeking decisions fully if they are interviewed as close as possible to the time they were experienced, and preferably before their diagnosis is known.

Our aim was to understand the symptom evaluation, or ‘appraisal’, and help-seeking decisions of patients with symptoms suggestive of lung cancer. In this paper, we report the results from an interview study that recruited people with respiratory symptoms referred to specialist respiratory services for consideration of possible cancer, irrespective of their subsequent diagnosis. This method enabled us to explore the complex processes and events that shaped patient appraisal and help-seeking from when they first noticed a symptom, to first consultation with a healthcare professional (HCP), through until they were referred.

## Methods

### Design and definitions

This in-depth, face-to-face interview study was nested within the SYMPTOM Lung Study (http://discovery-programme.org/symptom_study.php). The SYMPTOM study was a prospective cohort study investigating associations between symptoms and other factors on the total diagnostic interval and stage of diagnosis among patients with symptoms suggestive of lung, colorectal and pancreatic cancer. This interview study used qualitative methods to explore the factors that affected patient appraisal and help-seeking for respiratory symptoms. Ethical approval was obtained from Cambridgeshire 3 Research Ethics Committee (10/H0306/50).

**Figure 1:**
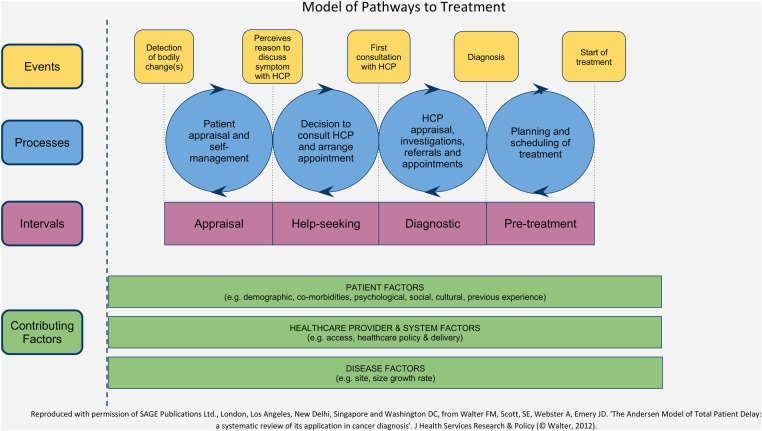
Model of pathways to treatment (HCP, healthcare professional).

The study design, including data collection and analysis, was underpinned by the theoretical approach of the Model of Pathways to Treatment (figure 1).^23^ ^24^ The model enables explicit consideration of patient, disease and healthcare factors that impact on patients’ appraisal of symptoms and decisions to seek help. Using a theoretical framework and the definitions of events along the patient pathway reflects best practice as defined in the Aarhus Statement.^25^ Detecting bodily change, perceiving a reason to seek help and first consulting a HCP are key ‘milestones’ or events in the pathway to treatment, and represent the ‘time to presentation’ (TTP).^25^ We define TTP as the interval between the patient-reported date of first noticing a symptom and their first consultation with an HCP, usually their GP. However, as one-third of patients with lung cancer consult their GP three times before referral,^26^ for participants who are not referred after the first consultation, we have also defined their further symptom appraisal and decisions to seek help again as the ‘Re-appraisal Interval’. This definition has resonance with the iterative nature of the Appraisal and Help-seeking Intervals as illustrated in the Pathways to Treatment model, figure 1.

### Study setting and recruitment

Recruitment to the study was undertaken when patients were referred to specialist respiratory clinics in secondary and tertiary care at five hospitals in the East and North East of England. Patients aged 40 years and over, referred to hospital via urgent (2 week wait), routine and diagnostic routes, for whom GPs had reported symptoms potentially suggestive of lung cancer, were mailed an introduction letter and SYMPTOM questionnaire, and invited to join the SYMPTOM study. They were also invited to take part in this interview study. We purposively sampled participants by region, selecting for variation of age, gender, educational level and diagnoses (cancer/other non-cancer).

### Data collection

Interviews were undertaken by NH, KM and LB between November 2011 and January 2013. Interviews were conducted as soon as possible after referral to specialist respiratory care, the majority within 10 weeks. Twelve participants were unaware of their diagnosis at interview (2 lung cancer). All diagnoses were confirmed from review of secondary care medical records.

Interviews were preceded by an explanation of the research process and signing of a consent form; consent was also rechecked at the end of each interview. We then used open-ended questions to explore the participant's appraisal of symptoms and help-seeking decisions with questions developed from our experiences of undertaking similar interviews with people recently diagnosed with cancer.^20^ Concepts explored included description and appraisal of the initial symptom/s and any self-care; the appraisal of subsequent symptom/s and the trigger/s that prompted consultation with an HCP; and the outcome of this consultation, and any further appraisal and help-seeking decisions. We probed the participant's understanding of symptoms, previous knowledge and understanding of lung cancer, and social influences on their decisions. A specifically developed calendar landmarking instrument was used to assist with participant recall, which helped to clarify the sequence and dates of events and intervals during their pathway to diagnosis.^27^

Interviews lasted between 40 and 65 minutes and were usually conducted in the participant's home; one person chose to be interviewed in university offices and another in their workplace. In several of the interviews a relative, usually spouse, was present. Relatives contributed to the interview by confirming participant's comments or adding detail to the account. Interviews continued until saturation of data, when no new themes were identified in three consecutive interviews.^28^ Audio-recordings of interviews were professionally transcribed verbatim and anonymised.

### Analysis

Analysis was an iterative process that started after the first few interviews. Framework analysis methods were used to ensure a systematic and rigorous progression through the five analytic steps: familiarisation with data; developing thematic framework; indexing data to framework; mapping and questioning the data; and theoretical interpretation.^29^ The thematic framework was developed by NH and KM in consultation with FW and LB; our study patient representative (MJ) also contributed to all stages of analysis. Data management was assisted by NVivo V.9.

During the mapping and questioning of data, we examined transcripts of participants diagnosed with lung cancer and compared them with those who presented with similar symptoms but who were diagnosed with other non-cancer conditions. We explicitly considered symptom appraisal, decisions about help-seeking, re-appraisal and further help-seeking. Following thematic analysis, data were charted by patient characteristics (age, gender, smoking, lung comorbidity and geographical region) to seek patterns or non-confirming cases between those diagnosed with lung cancer and those diagnosed with other non-cancer conditions.

## Results

### Patient characteristics

Seven hundred and seventy-six (81% n=963) of the participants recruited into the main SYMPTOM Lung study expressed an interest in taking part in the interview study. We undertook purposive sampling among this group, and only seven people declined to be interviewed because they were feeling unwell or were too busy with hospital appointments.

We undertook 35 interviews; 17 participants were diagnosed with lung cancer and 18 with other conditions. Table 1 shows the sociodemographic characteristics of the cohort, as reported in the SYMPTOM Lung study. The characteristics of the interview study cohort were similar to those of the SYMPTOM Lung study, although there were more smokers in the interview study. We purposefully sampled for people with a cancer diagnosis. In the interview study nearly half (49%) of the cohort had lung cancer compared with only 16% in the questionnaire study. Both cohorts had similar sociodemographic characteristics (ethnicity, education, employment). In the interview study nine reported they had existing lung comorbidity (4 COPD, 3 asthma, 2 other lung conditions).

**Table 1:** Participant characteristics

Diagnosis	Lung cancer N=17	Other conditions* N=18	Interview study N=35	SYMPTOM Lung study N=963
Location (England)				
East	8	8	16	
North East	9	10	19	
Sex				
Female	6	9	15 (42.9%)	441 (45.8%)
Male	11	9	20 (57.1%)	522 (54.2%)
Age mean years (range)	69 (57–83)	61 (41–88)	65 (41–88)	66 (40–95)
Ethnicity				
White	17	16	33 (94.3%)	931 (96.7%)
Other	–	2	2 (5.7%)	32 (3.3%)
Smoking				
Current smoker	5	4	9 (25.7%)	115 (12.2%)
Ex-smoker	10	6	16 (45.7%)	517 (54.8%)
Never smoked	2	8	10 (28.6%)	311 (33.0%)
Education				
Up to higher education	10	13	23 (65.7%)	655 (68.0%)
Higher education or equivalent	7	5	12 (34.3%)	308 (32.0%)
Employment†				
In employment	2	6	8 (23.5%)	274 (30.4%)
Disabled or unemployed	2	4	6 (17.6%)	56 (6.2%)
Retired	12	8	20 (58.8%)	570 (63.3%)
IMD quintile‡				
Least deprived 1	5	6	11 (31.4%)	317 (32.9%)
2	4	3	7 (20%)	216 (22.4%)
3	2	3	5 (14.3%)	160 (16.6%)
4	2	4	6 (17.1%)	126 (13.1%)
Most deprived 5	4	2	6 (17.1%)	143 (14.9%)
Number of GP appointments§				
1	9	7	16	
2 or more	6	10	16	
Type of referral to specialist services¶				
Urgent (2WW)	14	8	22	
Routine	0	8	8	
Comorbidities				
Respiratory				
COPD	1	3	4	
Other lung conditions	1	4	5	
Other				
Previous cancer**	2	1	3	
Diabetes	3	1	4	
Heart disease	4	4	8	
Arthritis	3	7	10	
Anxiety/depression	3	2	5	
IBS	2	2	4	

*Other conditions: pneumonia (3), COPD (2), asthma (1), pleurisy (1), fibrosis asbestosis (1), eosinophilic bronchitis (1), allergic rhinitis (1), acid reflux (1), calcified lung lesion (1), weight gain (1), post-infection sensitivity (1), nil abnormal found (3) and unreported (1).

†Missing data interview study n=1: questionnaire study n=63.

‡The IMD is a key data set on deprivation. The Indices measure levels of deprivation across seven distinct domains: Income Deprivation, Employment Deprivation, Health Deprivation and Disability, Education Skills and Training Deprivation, Barriers to Housing and Services, Living Environment Deprivation and Crime.

§Three participants referred following MRI or CT for other conditions.

¶5 Missing: missing data=3; referred through secondary care hospitals to tertiary centres=2.

**Previous cancer sites: kidney (1) and bowel (2).

COPD, chronic obstructive pulmonary disease; GP, general practitioner; IBS, irritable bowel syndrome; IMD, Index of Multiple Deprivation.

Of the 17 participants diagnosed with lung cancer, one had a lung metastasis from a primary colon cancer; eight had disease staged with potential for treatment with curative intent (stage I n=4; stage II n=4), while eight had more advanced disease (stage III n=1; stage IV n=7). The 18 participants with other conditions mainly had inflammatory conditions, see table 1.

### Duration of respiratory symptom/s

Seven participants did not seek help because of respiratory symptom/s; however, they did retrospectively recall respiratory symptoms and these data were included in our analysis. Three were referred to respiratory clinics following CT scan or MRI for non-respiratory symptoms, which reported lung abnormalities (2 lung cancer), and four had their respiratory condition opportunistically detected during consultations with GPs for other health concerns (2 lung cancer).

Most of the remaining 28 participants could recall when they first noticed their symptom/s and the date they went to the GP, though many were unable to recall the exact date they decided to seek help (ie, the start of the Help-seeking Interval in figure 1). We report the Appraisal Interval and first Help-seeking Interval as the TTP. The TTP ranged from 2 to 334 days; just under half consulted within 30 days of first noticing a symptom. Table 2 presents the characteristics of participants in the quartiles with the shortest and longest TTP, demonstrating similarities in age, gender, initial symptom attributions and trigger for help-seeking between those diagnosed with lung cancer and those diagnosed with other non-cancer conditions.

**Table 2:** Comparison of attribution and symptom/s that triggered help-seeking between participants with the shortest and longest quartile in the interval from first noticing a symptom to first presentation (ordered by first TTP)

TTP (days)	Sex	Age	Smoking status	Comorbidity	Dyspnoea	Haemoptysis	Dry cough	Productive cough	Chest pain	Back pain	Tightness in chest	Increased tiredness	Initial patient attribution	Lung cancer
Time between noticing a system and seeking help—shortest quartile														
2	F	45–49	Never	Asthma, IBS	●			○					Sinus infection	
7	M	65–69	Never	Arthritis						●			Gallstones	
7	F	80–84	Ex	–	●								Flu	
7	F	65–69	Ex	–	○		●				●		Chest infection	✓
14	F	50–54	Ex	Anxiety depression				●					Chest infection	
14	M	70–74	Ex	–	●								Getting older	✓
15	F	55–59	Current	–		●		●					Chest infection	
21	M	65–69	Ex	–						●			Muscle pain	✓
Time between noticing a symptom and seeking help—longest quartile														
49	M	80–84	Current	–	●	○							Getting older	✓
49	M	60–64	Ex	–					●				Muscle pain	✓
60	F	55–59	Never	–			●						Allergy	
58	M	60–64	Ex	–	●								Over exertion	✓
60	M	55–59	Current	–	●					○			Cardiac problems	
120	F	40–44	Never	–	○		●				○	○	Chest infection	
150	M	65–69	Ex	Heart disease				●				○	Cancer	✓
180	M	80–04	Ex	Heart disease, diabetes		●							Asbestos in lung	

● Indicates symptom/s that triggered help-seeking. ○ Indicates symptom/s reported that did not trigger help-seeking.

F, female; M, male; IBS, irritable bowel syndrome; TTP, time to presentation.

For the 16 (6 lung cancer) who consulted their GP two or more times before referral to specialist respiratory services, we also report the number of days between their first consultation and their referral: the Re-appraisal Interval. The time between first consultation and referral was between 10 and 182 days; for seven participants it was over 60 days (2 lung cancer). There were similarities in the Re-appraisal Interval between participants diagnosed with lung cancer and those diagnosed with other non-cancer conditions, although overall, patients diagnosed with lung cancer had fewer GP visits and were mainly referred under the 2 week wait referral system (table 3).

**Table 3:** Re-appraisal Interval: time between first noticing a symptom to first consultation and first consultation until referral to specialist respiratory services, by time intervals (ordered by first TTP)

	Time from noticing a symptom to first consultation (TTP) (days)	Time from first consultation to referral (Re-appraisal) (days)	Number of consultations	Type of referral	Gender and age	Diagnosis or lung cancer staging (T N M)*
Diagnosed with cancer						
1	3	60	2	Urgent	M, 75–79	IB 0 0
2	7	14	2	Urgent	M, 75–79	IV 2 IA
3	21	14	2	Urgent	M, 65–69	IV 2 IA
4	49	35	2	Urgent	M, 80–84	IIA I 0
5	50	102	4	Urgent	F, 70–74	IV 3 IA
6	90	133	4	Urgent	F, 55–59	IIB 0 0
Diagnosed with other conditions						
7	2	28	3	Routine	F, 45–49	Nil abnormal detected
8	2	35	4	Routine	F, 50–54	Emphysema
9	7	182	3	NA	F, 80–84	Interstitial pneumonia
10	14	80	3	Routine	F, 50–54	Asthma
11	15	63	2	Urgent	F, 55–59	Emphysema
12	30	138	4	Urgent	F, 65–69	Eosinophilic bronchitis
13	60	10	2	Urgent	M, 55–59	Tracheobronchitis and GORD
14	60	34	3	NA	F, 55–59	Seasonal allegoric rhinitis
15	60	60	2	Routine	M, 55–59	COPD
16	120	126	4	Routine	F, 40–44	Erythema suggestive of GORD

*T=tumour size; N=Regional lymph; M=Distant metastasis; Stage of cancer is indicative of whether treatment will be curative in intent.

COPD, chronic obstructive pulmonary disease; F, female; GORD, Gastro-oesophageal reflux disease; M, male; NA, not applicable; TTP, time to presentation.

### Qualitative themes

We found many similarities in the appraisal and help-seeking of participants who were diagnosed with lung cancer compared to those diagnosed with other conditions. Only two participants reported that they immediately thought of cancer because of their symptoms (1 lung cancer). We report the data in three sections:

- *The appraisal of symptoms* describes how they are difficult to recognise, and that lung comorbidities often masked respiratory changes, the alternative explanations offered for the cause of symptoms, and the ways in which symptoms were self-managed prior to help-seeking.
- *Deciding to seek help* describes the factors that helped to endorse a decision to seek a GP consultation, including symptoms such as signs of acute illness, recognising a changing symptom, symptoms not responding as expected, the influence of family members and the social network, increased awareness of lung cancer and the impact of smoking on help-seeking.
- *Symptom re-appraisal and returning to the GP* describes the factors that shaped the decision to seek a further consultation including symptom change, increasing impact of symptom on activities, increasing concern about personal risk of having lung cancer and the role of the GP in endorsing such help-seeking.

Extracts from interviews illustrate the results; each quotation is contextualised by the participant's gender, age band, smoking history, TTP in days, diagnosis (cancer, non-cancer) and if diagnosis was not known at time of interview.

### The appraisal of symptoms

The first interval in the patient pathway is the appraisal and self-management of symptom/s. If a symptom was difficult to recognise, attributed to ageing or smoking, or appraised as a condition that could be self-managed, participants often remained for some time in the appraisal interval. We found no evidence of differences between those diagnosed with lung cancer and those diagnosed with other non-cancer conditions across any of the factors, as shown in table 4.

**Table 4:** Time to Presentation (TTP): factors that impacted on symptom appraisal and initial help-seeking across people diagnosed with lung cancer and those diagnosed with other conditions

	Participants diagnosed with lung cancer	Participants diagnosed with other conditions
Factors that increased timeliness of help-seeking		
Symptom sign of acute illness	*You do the usual, you take your Lemsip's and your Beechams Powders and when it doesn't clear up after a week you think well you need some antibiotics or something slightly stronger. So that was when I went to the doctors* (M, 75–79, smoking not recorded, 7 days)	*I went to the doctor actually quite quickly because I had to fly and I knew that I would have trouble in the aeroplane if I didn't get some medication* (F, 50–54, ex-smoker, 14 days)
Symptom caused concern	*I went up a slight slope, and when I got to the top I was absolutely panting and really short of breath. … And I sat till I got my breath back, then I carried on normally, but when I got back home, on thinking about it, it wasn't right, and so that was the trigger point* (M, 70–74, ex-smoker, 15 days)	*I found myself very short of breath the onset was very quick and I thought ‘I don't think I should feel like this’, and I thought if I'm puffing like this rang the surgery and said ‘do you think you could make me an appointment for the next day because I'm really short of breath* (F, 80–84, ex-smoker, 7 days, diagnosis not known)
Influence of family	*Wife persuaded me to go to the doctor about it. I wasn't too worried about it, it wasn't significant to me* (M, 75–79, ex-smoker, 10 days, diagnosis not known)	*It didn't get any better, it got worse. So (daughter's name) would say, mother, that cough is awful and husband was saying the same* (F, 65–69, never smoked, 30 days, diagnosis not known)
Factors that prolonged TTP		
Competing responsibilities	*I was so damn busy, too busy to think of this…I was busy with all sorts of things, work and everything, and eventually I made the appointment* (F, 70–74, ex-smoker, 50 days)	*It started about the end of October in Australia and I saw my doctor about, the Monday before Christmas, … the cough had got worst whilst I was away* (F, 55–59, never smoked, 60 days)
Misattribution of symptoms	*I thought it was my asthma getting worse because I was getting more breathless I honestly truly believed it was my asthma getting worse* (F, 55–59, ex-smoker, 90 days)	*Breathlessness 2 or 3 months, but I mean it's just something which is coming on, so you don't get over concerned* (M, 55–59, smoker, 60 days, diagnosis not known)
Healthcare factors	*I didn't go because I couldn't explain it. I thought I don't want to waste his time just saying “I've, somehow I feel a bit strange” or whatever it is* (M, 75–79, ex-smoker, 56 days)	*Whenever I'm out of this situation (immigrant visa expired) then I will go and you know talk to the GP about it* (M, 40–44, never smoked, 334 days, diagnosis not known)

F, female; M, male; TTP, time to presentation.

#### Difficult to recognise

The insidious nature of some respiratory symptoms made it difficult to recognise a change in respiratory function “breathlessness has been creeping up” (M, 55–59, smoker, 60 days, non-cancer). An elderly man diagnosed with lung cancer explained he felt “not quite right, yucky, bit weary and tired”. He said he could not describe his symptoms sufficiently to justify visiting the doctor and waited 8 weeks before consulting his GP.

Having a lung comorbidity, such as COPD, could make it difficult to notice a change in symptom/s and some participants were only alerted to their respiratory symptom during consultations for other conditions:

I've always been breathless for 30-odd, it's 34 years since I got this heart disease, then I have COPD and emphysema. (M, 55–59, smoker, opportunistically detected, cancer)

Seven participants had not sought help for their symptoms although, in retrospect, those diagnosed following a CT scan or MRI for another reason, could often recall respiratory symptoms. These symptoms had not caused them concern as this woman diagnosed with lung cancer said about her cough:

P. I suppose it's been going on a while

Int. Would that be a few weeks or less than that?

P. Oh, more than that it was a dry tickly cough

Int. Ok, have you had any breathlessness?

P. A little, but that could be for other reasons…had it not been picked up on the scan I still wouldn't have gone to the GP. (F, 65–69, never smoked, opportunistically detected, cancer, diagnosis not known)

#### Alternative explanations for respiratory symptoms

Alternative explanations provided for the cause of symptoms were usually contextualised by anticipated changes in bodily function, or as a reaction to specific activities. For example, older people may expect to be more breathless on exertion: “I noticed maybe a few time I was not able to walk as fast as I used to be able to do, but I didn't think much about it…I am 72 I am going to begin to slow down” (M, 70–74, ex-smoker, 14 days, cancer).

Not all smokers mentioned smoking when discussing their appraisal or help-seeking but the few who did explained their symptom could be due to smoking. Another explanation, particularly for pain, was muscle strain. The explanation was justified within the context of activity: “I do a lot of work around the garden, picking things up, obviously muscles go into a bit of a spasm, so not to worry” (M, 65–69, ex-smoker, 21 days, cancer).

#### Self-management of symptoms

During the appraisal interval some participants tried to contain and self-manage their symptoms. By drawing on their knowledge about the cause of similar symptoms, their judgements about the cause of their symptom influenced subsequent decisions to seek healthcare:

It started in February and I just thought a tickly cough, it just went on and on and I thought it was a cold and then I thought, perhaps it's the beginning of hay fever so I just kept leaving it, trying hay fever tablets. (F, 40–44, never smoked, 121 days, non-cancer)

An alternative explanation for self-managing the symptom was occasionally situated within a dislike of attending the doctor's surgery:

I don't particularly enjoy going to the doctors so I went to the chemist and gradually built up a line of medication from the chemist these cough bottle I decided I would take rather than going to the doctors. (F, 65–69, never smoked, 30 days, non-cancer, diagnosis not known)

### Deciding to seek help

The decision to consult an HCP and move from appraising symptoms to seeking help was triggered by patient, disease and, to lesser extent, healthcare factors. Appraising symptom/s as a sign of acute illness prompted timely help-seeking. Family members and friends recognised symptoms in the participants and encouraged them to seek help. Increasing awareness of the signs of lung cancer, usually due to the ‘Be Clear on Cancer Campaign’, prompted help-seeking. Participants did not report that their smoking behaviour was an inhibitor to help-seeking. Competing responsibilities and limited access to healthcare prolonged the TTP for only a few participants, see table 4.

#### Symptoms as a sign of acute illness

Just over half of the participants consulted their GP within 30 days of first noticing their symptom, usually when it was attributed to a chest infection, see table 4. Drawing on previous experience and knowledge they quickly sought a consultation anticipating a prescription to alleviate symptoms:

I thought it might be chest infection in which case you know, antibiotics and away you go. (F, 65–69, ex-smoker, 7 days, cancer)

I thought that I needed maybe some antibiotics if I'd got a chest infection. (F, 50–54, smoker, 2 days, non-cancer)

When acute illness was experienced help-seeking was rapid, and those diagnosed with cancer and those diagnosed with other non-cancer conditions both said they had been well before their acute symptoms. When asked to clarify how long she had been feeling breathless one woman explained “maximum a week, up until then was still swimming 3 times a week, I'd walk for a good hour every day, I'd play table tennis just carry on as normal” (F, 65–69, ex-smoker, 7 days, cancer).

Symptoms not appraised as being due to a lung condition were often also attributed to an acute illness based on previous experience. For example, one man with a history of intermittent pain in the lower back and recent gallstones attributed his pain to a kidney infection and sought help promptly.

#### Recognising a changing symptom

Having a lung comorbidity meant patients were already living with chronic respiratory symptoms yet they were still able to disentangle and identify the ‘unusual’ or changing symptom. Even when a symptom was distinct from what was normally experienced some undertook ‘watchful waiting’ to see if the change was repeated before seeking help:

I've got COPD but I never coughed up blood before. I thought it will clear up but after two weeks it didn't so I thought I had better get it checked. (M 70–74, ex-smoker, 14 days, non-cancer)

When other factors highlighted the change, such as the failure of self-management or usual treatment to control the symptom, then the need for timely help-seeking was reinforced:

It wasn't like my normal asthma cough, I'd use my inhaler it had no impact at all … still continue coughing. (F, 45–49, never smoked, 2 days, non-cancer)

#### Symptoms not responding as expected

When symptoms were appraised as being self-limiting and not requiring medical intervention, such as due to allergy or muscle strain, help-seeking was only instigated when the symptom continued or failed to respond in the expected way. A cough appraised as an allergy that did not improve when the seasons changed triggered an initial consultation.

A seasonal allergy… (then it's) December, it's freezing cold and I was continuing to cough so I rang the doctor. (F, 55–59, never smoked, 60 days, non-cancer)

Similarly, with an appraisal of muscle pain there was the expectation that the pain would subside. When this did not happen or the pain increased there was an urgency to seek help:

It (back pain) got worse and worse, absolute agony…we had a couple of nights on holiday and I thought if it is still aching when I get back I'll call the doctor…we actually got back at 3 in the morning and I actually managed to get an appointment that day. (M, 65–69, ex-smoker, 21 days, cancer)

#### Influence of family members and the social network

A persistent cough was reported by approximately a third of participants (7 lung cancer). The cough was usually publicly noticeable, for instance, the tickly cough which made it difficult to talk and led to retching, and the expectorant cough with sputum and tissues. Family members, friends and work colleagues readily commented on a persistent cough:

Our friends were saying “Oh you know that cough is dreadful… it's just going on and on.” (F, 55–59, never smoked, 60 days, non-cancer)

A friend said perhaps you ought to get that cough looked at. (F, 70–74, ex-smoker, 50 days, cancer)

Older participants tended to attribute increasing breathless to a normal part of aging and their help-seeking was also often prompted by relatives, illustrating the importance of raising awareness of cancer symptoms in the older population:

My daughter said “I've never walked with you and you've lagged behind…” then she started to question about this breathing. (M, 80–84, smoker, 49 days, cancer, diagnosis not known)

When symptoms were ignored by the participant, the family was proactive in endorsing and sometimes organising the first consultation:

Probably I've been ignoring it for a few weeks, you know… the wife took it out of my hands she phoned the doctor. (M, 55–59, smoker, 60 days, non-cancer, diagnosis not known)

Even when participants lived alone, those who spoke to family and friends about their symptoms were encouraged to consult a GP, “brother said ‘I'm telling you now to go to the doctor” (M, 70–74, ex-smoker, 14 days, non-cancer).

Another man who lived alone explained “my friend went up to see the doctor and said I was in a bad way and the GP came here” (M, 60–64, ex-smoker, internal hospital referral for sleep apnoea tests, non-cancer).

#### Increased awareness of lung cancer

A cough was often the symptom that triggered help-seeking. Public awareness of a cough as a sign of lung cancer was raised by the ‘Be Clear on Cancer’ Lung cancer campaign, which ran across England during the period of interviewing.^16^ Five participants had seen the information and they focused on the headline message about cough being a warning sign of cancer but could not recall the other symptoms. Two did not have a cough so did not feel the advert applied to them. One said it had raised their awareness of their cough and triggered their help-seeking, although it had not specifically heightened their concern of lung cancer:

Until that advert came on, I never really took much notice of this cough… then it dawned on me I had this cough for a couple of weeks, so I waited till the third week and I went. (F, 55–59, smoker, 15 days, non-cancer, diagnosis not known)

The remaining two reported that the information had little impact, as they had already made the decision to book a GP consultation. Furthermore, one reported the campaign had slightly prolonged their help-seeking:

Had that not been screened I would have been to the doctors the week before, because I was saying to myself, it's just ‘cos that's on you're getting worried, so it had the adverse effect. (M, 65–69, ex-smoker, 150 days, cancer)

#### Impact of smoking on help-seeking

Those who had a history of smoking did not initially seek medical help with a concern of possible lung cancer and being a smoker did not impact on TTP, as in table 2. Rather, the cancer risk implicit in smoking was considered more when participants reappraised their symptoms following the first consultation. Nonetheless, when there was a change in the smoker's cough the decision was made to seek help:

Because if you are a smoker, see first thing in the morning it gets you, well I was waking up through the night with this dry cough, but it never lasted long and that's when I really started ooh have I got cancer, like lung cancer. (M 55–59, smoker, 60 days, non-cancer, diagnosis not known)

### Symptom re-appraisal and returning to the GP

Participants were generally happy with the outcome of their initial consultation, but when the prescribed treatment was not effective or their symptom changed, they started a process of re-appraisal and made decisions about whether to seek another GP consultation. There were similarities in the help-seeking decisions of those diagnosed with lung cancer and those diagnosed with other non-cancer conditions (see table 5).

**Table 5:** Re-appraisal: factors that triggered further help-seeking following initial consultation across people diagnosed with lung cancer and those diagnosed with other conditions

	Participants diagnosed with lung cancer	Participants diagnosed with other conditions
Concern about symptom	*I thought it strange to have a muscular problem in my back that hadn't got better in a couple of weeks so I thought it might be something, not necessarily sinister, well I suppose a slipped disc or a bone problem* (M, 65–69, ex-smoker, 21 days, 2 GP visits 14 days)	*I coughed up a bright red blood clot. It was while I was waiting to see doctor (for a persistent cough), I phoned them up and I said, “Look, I need to see someone and I need to see someone today”* (F, 55–59, smoker, 15 days, 2 GP visits 63 days, diagnosis not known)
Increasingly affected ability to undertake activity	*I take the dog out every day and that was getting less and less, the walk, so I knew something was wrong and I was getting to the stage where I didn't want to take her* (F, 55–59, ex-smoker, 90 days, 4 GP visits 133 days)	*Coughing with the fan heater and that kind of dry heat and because of my job I go into hot houses and then back to my car, back to hot houses all the time and that set the coughing off* (F, 40–44, 120 days, 4 GP visits 126 days)
Increasing concern that symptom indicative of lung cancer	*I read an article in the paper about a man who had a cough for weeks, found he had lung cancer and I remember thinking ‘oh god’ have I got that* (F, 70–74, ex-smoker, 50 days, 4 GP visits 102 days)	*My husband decided I'd got lung cancer. (Laughs) Well a friend of ours had exactly these symptoms and had lung cancer* F, 55–59, never smoked, 60 days, 3 GP visits 34 days)
Increasing concern about candidacy for lung cancer	(Negative case) *I was expecting a clean bill of health, I mean I've never shown any sign of illness, I'm 75 now and apart from the odd bit of flu I've never really been ill all my life* (M, 75–79, smoking not recorded,7 days, 2 GP visits 14 days)	*Because of the potential for methotrexate to be a problem and the fact that I had had a cough for longer than 3 weeks* (F, 56, never smoked, 60 days, 3 GP visits 34 days, non-cancer) *I thought I am a smoker, I've been a smoker for a lot of years that (cancer) crossed my mind* (F, 55–59, smoker, 15 days 2 GP visits 63 days, diagnosis not known)
Further help-seeking endorsed by GP	*The doctor said it looks like being muscular I'll give you some painkillers and come back in couple of weeks if it hasn't gone. So two weeks exactly I went back* (M, 65–69, ex-smoker, 21 days 2 GP visits 14 days)	*The doctor asked me to go back in a fortnight which I did* (F, 81, ex-smoker, 7 days, 3 GP visits 182 days, non-cancer, diagnosis not known) *The doctor did say, try the steroids and the one inhaler to start with, see how you go, because you can come back* (F, 40–45, never smoked, 121 days, 3 GP visits 126 days)

F, female; GP, general practitioner; M, male.

In this section each quotation is contextualised by the participant's gender, age band, smoking history, TTP in days, re-appraisal time in days and diagnosis.

#### Symptom change triggering further help-seeking

Following their initial consultation, an awareness of symptom changes often triggered a further GP consultation. For some it was the recognition of new symptoms:

We all sort of joked about my cough, but no, I was seriously very, very tired, and I suddenly thought “My God, I feel I've aged five years in five months…” and I thought no, wait a minute, you are breathless as well. (F, 70–74, ex-smoker, TTP 50 days, Re-appraisal 102 days, cancer)

For others it was the persistence or an increase in the severity of one or more of their symptoms that made them decide to seek further help:

The coughing was worse and it started really hurting my lungs and I started really being nervous about it because every time when I coughed it felt like as if I was ripping my lungs apart, it was really painful. (F, 50–54, ex-smoker, TTP 14 days, Re-appraisal 80 days, non-cancer, diagnosis not known)

When the symptom was recognised as a warning sign, such as coughing up blood, the urgency to return to the GP increased for some but not all people (table 5). For example, if the blood in the sputum could be attributed to other causes, the urgency to seek further help was reduced, as illustrated by a man who was using inhalers following his first consultation:

I started to cough I noticed flecks of blood. I took no notice of it and thought possible it was the stuff I was breathing in, or that it is just a burst blood vessel in my lungs. (M, 80–84, smoker TTP 49 days, Re-appraisal 35 days, cancer)

#### Increasing impact of symptom on activities

During the re-appraisal, participants described how symptom/s increasingly curtailed their ability to undertake work and leisure activities. This increasing impact of symptoms on activities acted as a trigger to reconsult:

I was trying to go to the shop and it was getting to be a bit of an embarrassment because in the shop, if I coughed me eyes would water and I'd choke or be sick. So I'd have to leave me shopping, come out the exit, try to pull myself together, then go back in. (F, 65–69, never smoked, TTP 30 days, Re-appraisal 138 days, non-cancer, diagnosis not known)

#### Increasing concern at personal risk of lung cancer

During the process of re-appraisal of symptoms several participants became increasingly concerned their symptoms were indicative of lung cancer. Personal predisposition for lung cancer was considered within the context of family history and previous environmental exposure to carcinogenic materials, particularly smoking. Drawing on their knowledge of lung cancer from seeing relatives or friends with the disease, the match or mismatch in symptoms shaped their re-appraisal and subsequent decision to seek further help:

My dad was coughing sputum, he coughed a lot of sputum, but I haven't, so they were totally different. My dad had a cough, I didn't have a cough, so there was a lot of differences… so the furthest thing from my mind it was going to be lung cancer. (F, 55–59, ex-smoker, TTP 90 days, Re-appraisal 133 days, cancer)

I thought it was lung cancer ‘cos my mam died of lung cancer… she used to always have like a dry cough, and I was having a bit of a dry cough. (M, 55–59, smoker, TTP 60 days, Re-appraisal 60 days, non-cancer, diagnosis not known)

Heightened concern about personal risk for lung cancer due not only to smoking but also to environmental exposure seemed to facilitate further help-seeking:

I've been smoking all my life more or less, I did work with asbestos when I was an apprentice and you think well maybe this is what it is all about maybe you have got cancer. (M, 55–59, smoker, TTP 60 days, Re-appraisal 10 days, non-cancer, diagnosis not known)

#### GPs role in endorsing further help-seeking

The decision to reconsult was not always an easy one. A minority of participants reported they felt the GP was dismissive of their symptom/s and concerns, “I said no, no, you're not listening to me I've had this cough for ages and ages, it's not just an overnight thing” (F, 65–69, never smoked, TTP 30 days, Re-appraisal 138 days, non-cancer, diagnosis not known).

Explicit advice on when to return seemed to legitimise a further consultation even when new symptoms had not developed (table 5). However, the absence of advice on further monitoring of symptoms and the appropriateness of a further consultation led some to revert to self-managing symptom/s:

Doctor gave me some antibiotics and he said it would do the trick. It didn't… so I left it, I left it and I left it and I continued another line of cough mixtures and stuff. (F, 65–69, never smoked, TTP 30 days, Re-appraisal 138 days, non-cancer, diagnosis not known)

Management of comorbidities often led to regular contact with their GP, but it was apparent that opportunities to investigate symptoms suggestive of lung cancer were not always maximised.

Because I had the x-ray in July Dr didn't think it (pain) would be anything but we would keep an eye on it. …I was going to the doctors maybe once a fortnight, once a month and every time it was for something else and I forgot to mention it even though it was happening. (F, 55–59, ex-smoker, TTP 90 days, Re-appraisal 133 days, cancer)

Consultations for comorbidities were not only with the GP; symptoms suggestive of lung cancer were mentioned to other HCPs but there was little evidence of effective advice on symptom monitoring being provided to the participants:

I go six monthly to the nurse in the clinic and I mentioned to her I was spitting blood and she said “well make an appointment with the doctor” but I just went out and never bothered. (M, 85–89, ex-smoker, 180 days, non-cancer, diagnosis not known)

When this patient returned a few months later and reported his haemoptysis again the nurse phoned the doctor directly and he had a chest X-ray within 24 h.

## Discussion

### Main findings

We believe this is the first study to explore the appraisal and help-seeking decisions of patients responding to symptoms suggestive of lung cancer. Unsurprisingly, there are few differences in the appraisal of symptoms, decisions to seek help, TTPs and re-appraisal intervals between people diagnosed with lung cancer and other conditions. We found evidence of complex reasoning and decision-making supporting the processes of deciding whether and when to seek help. While patients often recognised new symptoms or subtle symptom changes, even against a background of the expected symptoms of lung and cardiac comorbidities or being a smoker, they often did not feel the need to seek help because they developed alternative explanations based on their previous experiences or believed they could self-manage the symptoms. Help-seeking was triggered by recognising the symptoms as signs of acute or serious illness, the progression or persistence of existing symptoms or new symptoms, the influence of family members and their social network, particularly due to the visibility of symptoms, and sometimes current public health messages. Half the sample received treatment for other conditions, such as for acute respiratory illness, and had not been referred after their first GP consultation. Symptom monitoring and re-appraisal followed; returning to the GP was again influenced by the progression or persistence of existing symptoms or new symptoms, an impact of symptoms on daily living activities, and increasing concern about underlying serious disease and cancer. We found little evidence that patients received adequate advice from their GPs about symptom monitoring or reasons to return for review.

### Strengths and limitations

The major strength of this study is that we interviewed people during their pathway to diagnosis and treatment, often before they received their diagnosis; therefore we were able to compare accounts of people diagnosed with lung cancer and other non-cancer conditions. We sought to interview people as early in their disease development as possible. Interviewing 12 people before they received their diagnosis helped to reduce post hoc rationalisation and recall bias,^9^ ^11–13^ and the remaining interviews were conducted as soon as possible after diagnosis (range: 1 day to 16 weeks), with 15 (43%) occurring within 4 weeks of diagnosis. Importantly, we were able to include people with advanced stage cancer before they became too ill to participate in research.

In accordance with the guidelines outlined in the Aarhus Statement on improving design and reporting of studies on early cancer diagnosis,^25^ we used a rigorous study design with the theoretical Model of Pathways to Treatment^23^ underpinning the interview schedule as well as the analysis. The calendar-landmarking instrument helped some participants recall dates and symptom changes. Purposive sampling from two areas of England ensured data were reported from people with differing socioeconomic backgrounds and differing exposures to carcinogenic environments. For example, some people in the North East spoke of personal risk due to proximity to, or employment in, heavy industry. Our broad range of scientific and clinical expertise helped ensure consensus in the findings, and we benefitted from the input of our lay member at all stages of the research process, including interpretation of the data.

We acknowledge that the experiences of patients from these two regions may not be representative of those from other regions of the UK and that we cannot know the experiences of those who did not take part, although the interview sample has similar demographic characteristics to the main SYMPTOM Lung study (see table 1). Furthermore, we are only able to report the perspectives of patients who had been referred through primary care and had consulted their GP, but we have not been able to access the appraisal or help-seeking experiences of those who presented first at accident and emergency department.^30^

While we used a calendar-landmarking instrument during the interviews to ensure, as far as possible, the accuracy of time intervals, some people were unable to recall precise dates and we took the dates from their responses to the questionnaire in the main SYMPTOM lung study. The nature of qualitative data collection is such that we can only report the experiences patients chose to divulge; it may be that they did not share experiences that they considered to be private or embarrassing.

### Comparison with existing literature

We recruited people with symptoms suggestive of lung cancer, and included patients who were not aware of their diagnosis at the time of interview, those who were diagnosed with other non-cancer conditions as well as people diagnosed with early and later stage lung cancer. We are therefore able to add new insights to the existing literature on the experiences and decision-making processes of people with lung cancer^9–13^ in order to develop new targeted interventions to promote timelier lung cancer diagnosis. We found that initial alternative explanations for symptoms were based on patients’ previous experiences, for example, a dry cough during summer was attributed to hay fever, and slight breathlessness in winter attributed to a chest infection. This is concordant with findings from a UK interview study of patients with operable and inoperable lung cancer, which reported that they minimised, normalised or misattributed symptoms.^12^ However, that study also reported their patients lacked agency in seeking help; in contrast, patients in our study tended to either adopt ‘watchful waiting’ to see if symptoms improved, or to self-manage symptoms with over the counter drugs.

Other studies interviewing people with lung cancer have highlighted that lung and cardiac comorbidities, such as COPD and asthma, can delay symptom appraisal and timely help-seeking for lung cancer.^9^ ^12^ We found that many patients, especially those with comorbidities, showed a complex and sophisticated ability to distinguish minor changes in respiratory function from pre-existing symptoms due either to their comorbidities or smoking habits. They quickly noticed either change in their normal symptoms or the effectiveness of usual medication. The recognition of changing effectiveness of medication is a novel finding and highlights the way in which patients draw on information from different experiences and events to make a judgement on whether or not a symptom requires medical care.^31^

The nature of cough has been discussed in the context of those with lung cancer^32^ and we add to this discussion by reporting the impact of cough on help-seeking prior to diagnosis. The very public nature of a chronic cough prompted members of the family and the wider social network to encourage and endorse help-seeking. The impact of cough on daily activities, such as talking on the phone and shopping, also triggered initial and subsequent help-seeking. Living alone has been reported as a factor in prolonging help-seeking,^9^ ^33^ but in line with an earlier interview study by Tod *et al*,^34^ we found that if the participant's symptom was observed by, or discussed with, family and friends, then timely help-seeking often took place.

As we interviewed patients during a national ‘Be Clear on Cancer’ lung cancer campaign^16^ we are able to comment on the possible impact of the campaign on patient's symptom appraisal and help-seeking behaviour. Some patients who had seen the campaign had a heightened awareness of respiratory symptoms, particularly a persistent cough; for them the campaign endorsed the need to seek help in line with findings from other studies reporting the impact of similar health campaigns.^17^ ^18^ However, in our study there were two patients who reported that an increased awareness had slightly prolonged their help-seeking as they were concerned they were over-reacting.

This is one of the first studies to explicitly explore patient processes during the time between first consulting a HCP, and further consultations leading to investigations and referral. During the Re-appraisal Interval participants continued to consider symptom change, severity and duration as triggers to return to the GP, but rarely reported receiving guidance from their HCP on symptom monitoring or when to reconsult. There were examples of substantial time intervals between GP consultations.^5^ Those who were given explicit advice on symptom monitoring and when to return to the GP (so-called safety netting),^35^ promptly sought further consultations enabling GPs to continue diagnostic investigations in a timely way. We found some examples where patients with comorbidities accessed regular healthcare but the HCP did not seem to use the opportunity to follow-up on previously disclosed symptoms suggestive of lung cancer.

Despite the evidence suggesting that people delay help-seeking due to fear of cancer, we found that patients were often unconcerned that symptoms may be indicative of lung cancer even when they had increased risk due to current or recent smoking habits.^20^ ^36^ ^37^ In our study, concerns about candidacy for lung cancer were only discussed within the context of reappraising symptoms and when alternative explanations failed to respond as expected to initial treatment.

### Implications for clinicians and policymakers

Our findings provide further evidence for targeted public health campaigns that are tailored to specific groups such as smokers and people with other chest conditions who may have difficultly detecting symptom change, focusing not only on recognition of new, changing and persistent symptoms but also on recognising reduced effectiveness of medications. The role of family and social networks in recognising and discussing a symptom, and then endorsing help-seeking, could contribute more prominently to public health initiatives to raise community awareness of appropriate help-seeking for timely diagnosis of lung cancer and other serious lung conditions.

The vast majority of people who seek help for respiratory symptoms will not have lung cancer but our findings indicate that those with cancer and other non-cancer diagnoses all undertake similar complex reasoning and decision-making when deciding whether and when to seek help. There is a need for further research into the ways in which people make these complex decisions around assessing the seriousness and severity of symptoms, the triggers to seek a medical consultation, and their explanations about which symptoms can be self-managed. Understanding the social context in which risks of ill health are assessed would provide more opportunities for the development of targeted and evidence-based interventions to promote timely help-seeking.

Brindle and colleagues recently questioned whether GP elicitation of normalised symptoms could reduce delay in lung cancer diagnosis.^13^ Our findings confirm that GPs appear to miss opportunities, particularly among people at higher risk such as smokers, ex-smokers and those with chronic chest conditions; this may be due to their gatekeeper role and current guidelines. While acknowledging that the majority of patients presenting with respiratory symptoms will not have lung cancer, there nevertheless remains a need for vigilance and a systematic application of safety-netting procedures such as explicit oral and written instructions detailing expected symptom progression over time; recognition of changing, persistent and new symptoms that should prompt a further appointment; and a specified follow-up time.^35^
